# A Protective Strategy to Counteract the Oxidative Stress Induced by Simulated Microgravity on H9C2 Cardiomyocytes

**DOI:** 10.1155/2021/9951113

**Published:** 2021-04-20

**Authors:** Simone Guarnieri, Caterina Morabito, Michele Bevere, Paola Lanuti, Maria A. Mariggiò

**Affiliations:** ^1^Department of Neuroscience, Imaging and Clinical Sciences, University “G. d'Annunzio” of Chieti-Pescara, Chieti 66100, Italy; ^2^Center for Advanced Studies and Technology (CAST), University “G. d'Annunzio” of Chieti-Pescara, Chieti 66100, Italy; ^3^Department of Medicine and Aging Sciences, University “G. d'Annunzio” of Chieti-Pescara, Chieti 66100, Italy

## Abstract

Microgravity affects human cardiovascular function inducing heart rhythm disturbances and even cardiac atrophy. The mechanisms triggered by microgravity and the search for protection strategies are difficult to be investigated in vivo. This study is aimed at investigating the effects induced by simulated microgravity on a cardiomyocyte-like phenotype. The Random Positioning Machine (RPM), set in a CO_2_ incubator, was used to simulate microgravity, and H9C2 cell line was used as the cardiomyocyte-like model. H9C2 cells were exposed to simulated microgravity up to 96 h, showing a slower cell proliferation rate and lower metabolic activity in comparison to cell grown at earth gravity. In exposed cells, these effects were accompanied by increased levels of intracellular reactive oxygen species (ROS), cytosolic Ca^2+^, and mitochondrial superoxide anion. Protein carbonyls, markers of protein oxidation, were significantly increased after the first 48 h of exposition in the RPM. In these conditions, the presence of an antioxidant, the N-acetylcysteine (NAC), counteracted the effects induced by the simulated microgravity. In conclusion, these data suggest that simulated microgravity triggers a concomitant increase of intracellular ROS and Ca^2+^ levels and affects cell metabolic activity which in turn could be responsible for the slower proliferative rate. Nevertheless, the very low number of detectable dead cells and, more interestingly, the protective effect of NA, demonstrate that simulated microgravity does not have “an irreversible toxic effect” but, affecting the oxidative balance, results in a transient slowdown of proliferation.

## 1. Introduction

The presence of gravity on Earth deeply influenced the evolution of living beings, and during the past century, human space activities have opened new scientific challenges related to understand the effects caused by the low gravity environment (microgravity) on living organisms. Microgravity affects numerous features and functions of biological organisms, at the cellular as well as the organ or systemic level [1]. The permanence of astronauts in orbit to maintain satellites and at the International Space Station (ISS) has required attention on the effects induced by altered gravity on the human body. From this point of view, the effects on muscle and bone tissues have attracted considerable interest to develop countermeasures to attenuate musculoskeletal decline in space [2, 3]. Not less important, exposure to microgravity during space flight causes changes in the human cardiovascular system, including fluid shift, changes in systolic volume, and, after chronic exposure, a reduction of the left ventricle mass [4–6]. Clinical evaluations on astronauts during prolonged exposure to microgravity documented also very frequent alterations in the electrical assets of the heart, increasing the predisposition toward cardiac arrhythmias [7–9]. Even if some aspects of cardiac arrhythmias could be explained by sympathetic and vagal imbalance due to adaptation to microgravity, at cellular levels, it has reported also alterations in calcium handling that are potentially linked to contractile and arrhythmogenic dysfunctions [10, 11]. To date, very little is known about the mechanisms of cardiac remodelling induced by microgravity. During long-term exposure of mice to simulated microgravity (s-microgravity), it has reported an increase in ryanodine type 2 phosphorylation that is involved in cardiac remodelling, thus suggesting that abnormalities in calcium homeostasis could be also implicated [12]. Moreover, even if the cellular target(s) and the following events are still unclear, microgravity induces a stressful condition in the endoplasmic reticulum of neonatal rat cardiomyocytes. In this scenario, cardiac cells show an upregulation of mitochondrial proteins to preserve energy production and promote survival. This metabolic adaptation decreases protein translation and contributes to the development of cardiac atrophy [13]. Intracellular Ca^2+^ homeostasis in cardiomyocytes appears to be affected during space flight as well as during prolonged bed rest. Indeed, intracellular Ca^2+^ levels and patch-clamp measurements in cardiomyocytes from rat hearts after mechanical unloading showed a decrease in sarcoplasmic reticulum Ca^2+^ content and in the amount of Ca^2+^ released during the Ca^2+^-induced Ca^2+^ release process. Moreover, this reduction in the sarcoplasmic reticulum compartment is fully counterpoised by an increased influx of Ca^2+^ through L-type calcium channels [14]. The other face of the coin is represented by increasing evidences that microgravity could influence mitochondrial function and prooxidative conditions observed under real or simulated microgravity conditions. In many models, an increased level of reactive oxygen species (ROS) or the detection of products of oxidative reactions was shown, suggesting that oxidative substances increase during exposure to a microgravity-conditioned environment [15–18]. Studies coming from Cosmos 1887 experimentation highlighted morphological alterations in the rat heart tissue exposed to microgravity up to 12.5 days. Structural changes were observed not only in myofibrils but also at the level of mitochondria with reduction of their volume and breakdown [19]. Hindlimb unloading in mice, simulating a chronic exposure (21 days) to microgravity, showed alterations in the ratio between reduced and oxidized glutathione in cardiac tissue. After exposure, the glutathione content was reduced in cardiac tissue, while its oxidized form increased, indicating an increase in ROS production. Interestingly, in the same study, results from the late observation time points (up to 9 months after 21-days of hindlimb unloading) suggested that the heart had mostly recovered [20].

Even if numerous evidences regarding the presence of altered Ca^2+^ homeostasis and metabolic status with an oxidative imbalance were reported, there is no direct evidence about a possible interplay between intracellular Ca^2+^ and an overproduction of ROS in cardiomyocytes exposed to microgravity.

Based on these considerations, the aim of this study was to investigate the link between morphological and metabolic alterations induced by s-microgravity in a cardiomyocyte-like model with particular focus on the correlation between ROS production and intracellular calcium.

In this study, we used the random position machine (RPM) to simulate microgravity conditions and the H9C2 cell line as a cardiomyocyte-like model. The experimental plan was designed to reach the following main goals: (1) to verify the effect of s-microgravity on cell morphology and cytoskeleton architecture as well as cellular vitality, proliferation, and cycle; (2) to analyze the cell metabolic status mainly monitoring the intracellular Ca^2+^ levels and mitochondrial functions; and (3) to attempt a protective strategy to counteract the microgravity-induced effects.

## 2. Materials and Methods

### 2.1. Equipment and Cell Exposure Parameters

Microgravity conditions were simulated using a commercially available RPM connected to a control console through standard electrical cables (RPM ver. 2.0, Dutch Space, Leiden, The Netherlands). The main characteristics of this apparatus were previously described [21–23] and the use parameters reported in the manufacturer's manual. Mainly, the apparatus is a 3D clinostat consisting of two independently rotating frames, positioned one inside the other, thus exerting a net change in orientation on a biological sample mounted in the middle. This apparatus is a microweight simulator based on the principle of “gravity-vector averaging.” The desktop RPM we used was positioned within an incubator (to maintain the 37°C temperature and CO_2_ and humidity levels) and set in a 3D random mode with a mean angular speed of ±60°/s for both inner and outer frames during sample exposition [23]. The selected exposure times to s-microgravity on the RPM were 6, 12, 24, 48, 72, and 96 hours. These were identified considering the H9C2 cell proliferation and cycle timing in order to follow long-lasting biological events. Preliminary experiments were performed to test viable cell culture conditions and experimental reproducibility.

### 2.2. Chemicals and Materials

Unless indicated otherwise, the cell culture plastic ware was obtained from Becton Dickinson Falcon (Steroglass S.r.l., San Martino in Campo, Italy); the cell culture medium, sera, and antibiotics were purchased from Thermo Fisher Scientific (Monza, Italy); and the reagents and standards were obtained from Sigma-Aldrich (Milan, Italy).

### 2.3. Cell Cultures

H9C2 cell line, derived from embryonic BD1X rat hearts (CRL 1446; American Type Culture collection), was grown at 37°C under 5% CO_2_/air in Dulbecco's modified Eagle's medium containing (in mM) 2 glutamine, 18 sodium bicarbonate, 25 glucose, 1 sodium pyruvate, 100 units/ml penicillin, and 0.1 ng/ml streptomycin and supplemented with 10% heat-inactivated fetal calf serum. In selected experiments, the cells were treated with 1 mM N-acetylcysteine (NAC) as an antioxidant agent. NAC concentration was derived from data previously published [22, 24]; in addition before using NAC as antioxidant agent, we tested it on H9C2 cells to exclude its possible toxicity. Twenty-four hours after cell seeding, experiments were performed on cells cultured in the same incubator for up to 96 hours at 1 g or at s-microgravity in RPM. For the measurements of Ca^2+^, ROS, and O_2_^·−^ levels and of mitochondrial membrane potential, H9C2 cells were plated in special optic 96-well plates (cat. CLS3615 Corning Costar, Milan, Italy). For trypan blue exclusion tests, cytofluorimetric analyses, Western blots, analyses of glucose, and lactate levels, cells were plated in 35 mm Petri dishes. For immunofluorescence staining, cells were plated on glass slides fixed on petri dishes or *μ*-Dish 35 mm IBIDI imaging dish (IBIDI GmbH, Martinsried, Germany). All cell culture holders (microplates, dishes, etc.) were completely filled with culture medium and placed at 1 g or RPM culture conditions to avoid air bubbles and to minimize liquid flow; thus, the effects of both buoyancy and shear stress during rotation were negligible.

### 2.4. Viability and Proliferation Assays

The trypan blue exclusion assay was performed by staining the cells with a trypan blue dye solution (0.5% in phosphate-buffered saline (PBS)), and the stained cells were counted using a Bürker chamber. Blue-stained cells were considered nonviable.

### 2.5. Flow Cytometry Analysis

Cell cycle investigations were performed according to published protocols [25]. Control or s-microgravity exposed cells were washed with PBS, harvested with trypsin/EDTA, and centrifuged (500xg, 5 min). Cell pellets were fixed with 70% (*v*/*v*) ethanol and stained with a propidium iodide solution (50 *μ*g/mL propidium iodide and 100 *μ*g/mL RNAse in PBS, Sigma-Aldrich). The fluorescent intensity was recorded using a FACS Canto II flow cytometer (Becton Dickinson Biosciences, Franklin Lakes, NJ, USA). Samples were cultured in triplicate, and a minimum of 10000 events were acquired for each pellet. The percentages of cells in G0/G1, S, and G2/M phases of the cell cycle and apoptotic and necrotic populations were calculated using FlowJo software v8.8.6 (TreeStar, Ashland, OR, USA).

### 2.6. Immunofluorescence Staining

Cells were fixed with 4% paraformaldehyde in PBS for 10 min at 4°C and permeabilized with 0.1%Triton X-100 in PBS for 10 min, washed and treated with blocking solution (10% goat serum in PBS) for 1 h at room temperature. Cells were incubated with primary mouse antibody anti-*β* tubulin (1 : 50 dilution in PBS containing 10% goat serum, Thermo Fisher Scientific, Monza, Italy) overnight at 4°C followed by 1 h incubation at RT with the secondary antibody (goat anti-mouse AlexaFluor-488, 1 : 200 dilution, Thermo Fisher Scientific). After washing, the cells were stained with Alexa Fluor 546 Phalloidin (1 : 50 dilution, Thermo Fisher Scientific) for 1 h at RT, and the nuclei were counterstained with DAPI (10 *μ*M, Thermo Fisher Scientific) for 10 min at 37°C. For mitochondrial morphology, the cells were permeabilized, fixed, and blocked as reported above and incubated with primary rabbit antibody anti-TOM-20 (1 : 100 dilution in PBS containing 10% goat serum, Thermo Fisher Scientific) overnight at 4°C. The primary antibodies were revealed by 1 h incubation with goat anti-rabbit AlexaFluor-488 (1 ∶ 200 dilution, Thermo Fisher Scientific) at RT, and nuclei were stained with DAPI (10 *μ*M, Thermo Fisher Scientific) for 10 min at 37°C. For each sample, images were acquired from 5 different randomly selected fields of 3 independent experiments, using a Zeiss LSM800 URGB confocal microscope equipped with an upright AxioObserver Z1 microscope and a 40X N.A. 1.3 oil immersion objective and ZEN Blue 2.1 software (Carl Zeiss, Jena, D). Z scan acquisitions were performed at a resolution of 512 × 512 pixel, acquiring the focal planes every 0.420 *μ*m used as the starting point glass of the adherent side of the cells, and as the end point, the plane where the cells vanished from the acquisition field.

### 2.7. Quantitative Analyses of Immunofluorescence Staining

#### 2.7.1. Cell Height

Cellular height was calculated off-line using ZEN Blue 2.1 software and 3D-reconstructions of orthogonal projections (XZ) obtained from the Z-stack image acquisition. For each single cell, measurements were performed tracing the distance between the cell adhesion plan and the highest point of the cell body, often in proximity of the nuclear region.

#### 2.7.2. Cytoskeleton Architecture and Mitochondrial Network

Confocal fluorescence images were analyzed off-line using FIJI distribution of ImageJ (ver1.52p) [26]. To measure cytoskeletal organization, optical sections derived from the equatorial plane were processed to obtain a simplified morphological model using the “make binary” and “skeletonize” functions. On the processed images, the function “Analyze Skeleton (2D/3D)” calculated the averaged *β* tubulin or actin filament length (expressed as arbitrary units (a.u.)) in a cell.

The mitochondrial network morphology was analyzed in optical sections derived from the equatorial plane using MiNA, a macro tool for ImageJ developed by Valente and colleagues [27]. In samples at 1 g or s-microgravity, “individuals” (defined as the counts of the number of objects in the image that do not contain a junction pixel, it determines the number of punctuate or rod-shaped mitochondria), “branched structures in a single cell” (outlining the number of objects in the image that contain at least 1 junction pixel and are thus comprised of more than one branch, it defines the number of mitochondria networks inside the cell), and “mean branching length in single cell” (the average length of all rods/branches per single cell) were measured.

### 2.8. Western Blotting

Cells were scraped and collected in sample lysis buffer (62.5 mM Tris-HCl, pH 6.8, 2% SDS, 10% glycerol, 0.1 M dithiothreitol, and 0.002% bromophenol blue). Protein content was determined using a protein assay kit (Bio-Rad DC; Bio-Rad Laboratories Srl, Milan, Italy). Cell extracts were separated on 7.5% or 10% (*w*/*v*) homogeneous slab gels (40 *μ*g of protein/lane) using SDS-PAGE and then transferred to nitrocellulose membranes (Protran; Whatman-GE Healthcare, Milan, Italy). Membranes were hybridized with a mouse monoclonal anti-*β* tubulin antibody (1 : 500 dilution, Thermo Fisher Scientific) or mouse monoclonal anti-*β* actin antibody (1 : 1,000 dilution, Santa Cruz Biotechnology Inc., Heidelberg, Germany) followed by an incubation with horseradish-peroxidase-conjugated anti-mouse or anti-rabbit IgGs (1 : 10,000 dilution, GE Healthcare, Cologno Monzese, Italy). The relevant proteins were detected using chemiluminescence kits (Pierce EuroClone S.p.A., Pero, Italy), and the signals were acquired and analyzed using an image acquisition system (Uvitec mod Alliance 9.7, Uvitec, Cambridge, UK). A mouse monoclonal anti-GAPDH antibody (1 : 10,000 dilution, Merck S.p.A., Vimodrone, Italy) was used as a loading control.

### 2.9. Measurements of Glucose and Lactate Levels in Cell Culture Medium

Glucose and lactate levels in the growth medium were detected using a Free Style Optium glucometer (Abbot Laboratories, Rome, Italy) and a Lactate Pro Analyser (Arkray Inc. Kyoto, Japan), respectively.

### 2.10. Fluorescence Measurements in Live Cells

Cells were incubated with a normal external solution (NES in mM: 140 NaCl, 2.8 KCl, 2 mM CaCl_2_, 2 MgCl_2_, 10 glucose, and 10 HEPES, pH 7.3) containing one of the probes reported in Table 1 (Thermo Fisher Scientific) for 40 min at 37°C. After rinsing, the fluorescence signal was acquired at 25°C using a microplate reader (Synergy H1 multimode, Biotek, Bad Friedrichshall, Germany) and stored on a computer using the software GEN5. (Biotek, Bad).

Excitation and emission filters were set according to the specific probe used (Table 1). The fluorescence values of Fluo-4-, H_2_-DCFDA-, or MitoSox RED-loaded cells are expressed as the means (±standard errors of the means (SEM)) of *f*/cell number, where *f* is the fluorescence value acquired and normalized to the number of cells in the well. Fluorescence values of JC1-loaded cells are expressed as the means (±SEM) of the red/green fluorescence ratio, which depends on the mitochondrial membrane potential [28]. For each experimental condition, eight repetitions were performed in three independent experiments.

### 2.11. Cellular Oxidative Status and Damage

#### 2.11.1. Total Antioxidant Status

The total antioxidant status of cell populations was detected using the 6-hydroxy-2,5,7,8-tetramethylchroman-2-carboxylic acid- (Trolox-) equivalent antioxidant capacity (TEAC) assay. Cells sonicated in cold buffer (5 mM potassium phosphate, pH 7.4, 0.9% NaCl, 0.1% glucose) and centrifuged at 10,000xg for 15 min at 4°C were assayed as described by the manufacturer (Cayman Chemical, Ann Arbor, MI, USA). Briefly, 10 *μ*L of each sample containing equivalent amounts of cytosolic proteins, determined using the Bio-Rad protein assay (Bio-Rad Laboratories Srl), was added in triplicate to 10 *μ*L of met-myoglobin and 150 *μ*L of the chromogen solution supplied by the manufacturer. Reactions were initiated by the addition of 40 *μ*L of H_2_O_2_. After 3 min incubation at RT, the reaction mixtures were read at 750 nm using a microplate reader (Synergy H1 multimode, Biotek). Results were expressed as Trolox equivalents by reference to a linear calibration curve computed from pure Trolox-containing reactions (range 0–0.33 mM) as also previously reported [28].

#### 2.11.2. Protein Carbonyl Measurement

Protein carbonyl content, a marker of protein oxidation, was assayed using a protein carbonyl assay kit (Cayman Chemical). Briefly, the cells were rinsed with PBS, sonicated in 10 volumes of a buffer solution containing 50 mM MES (pH 6.7) and 1 mM EDTA, and processed as follows. After centrifugation (10,000xg, 15 min at 4°C), 200 *μ*L of cytosolic fraction was added to 800 *μ*L of 2,4-dinitrophenylhydrazine (DNPH) or 2.5 M HCl (blank control) and incubated at RT for 1 h in the dark. After the addition of 1 mL of 20% trichloroacetic acid (TCA) and incubation on ice for 5 min, the samples and blanks were centrifuged (10,000xg, 10 min at 4°C) and the resulting pellets were resuspended in 1 mL of 10% TCA and recentrifuged (10,000xg, 10 min at 4°C). This step was repeated three times using 1 mL of the ethanol : ethyl acetate mixture (1 : 1) to resuspend the pellets. The last protein pellets were resuspended in 500 mL of guanidine hydrochloride and centrifuged (10,000xg, 10 min at 4°C). The carbonyl content was determined based on the supernatant absorbance at 370 nm using a molar adsorption coefficient for DNPH of 22,000 M^−1^ cm^−1^. Results were expressed as nanomoles of DNPH per milligram protein, as previously described [28].

#### 2.11.3. Malondialdehyde Measurement

Malondialdehyde (MDA) forms, markers of lipid peroxidation, were analyzed using the OXItek TBARS assay kit (ZeptoMetrix Corp., Buffalo, NY, USA) following the manufacturer's instructions. Briefly, 100 *μ*L of SDS was mixed with 100 *μ*L of samples obtained from sonicated cells (in PBS), and 2.5 mL of thiobarbituric acid buffer reagent. Samples were then incubated at 95°C for 1 h, and the reactions were stopped by cooling in an ice bath for 10 min. After centrifugation (3000 rpm, 15 min), the supernatant absorbance was read at 532 nm. The amount of MDA was calculated by reference to a standard curve. Results were expressed as nanomoles of MDA per mg protein, as previously described [28].

### 2.12. Statistical Analyses

Experimental values are expressed as the means ± SEM. Statistical significance was assessed using Student's *t*-test with Prism5 software (GraphPad, San Diego, CA, USA). *p* values < 0.05 were considered statistically significant.

## 3. Results

### 3.1. Cell Morphology, Cytoskeleton Architecture, and Mitochondrial Network

**Figure 1:**
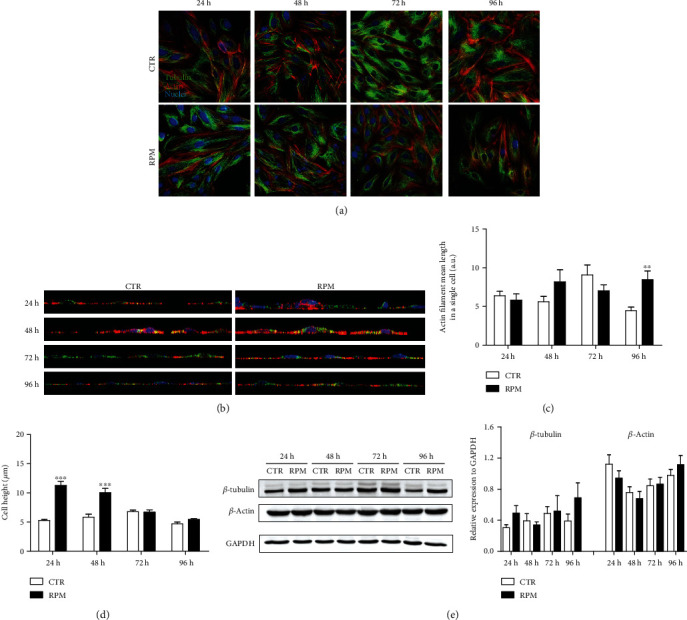
Cell morphology and cytoskeleton architecture. (a) Representative images of H9C2 cells at 1 g (CTR) or s-microgravity (RPM) stained for *β* tubulin (green), actin (red), and nuclei (blue) acquired at selected times (24, 48, 72, and 96 hours). Scale bar: 20 *μ*m. (b) Representative images of orthogonal XZ projections obtained from 3D reconstruction of Z-stack acquisitions. CTR (1 g) or RPM (s-microgravity) samples were stained to reveal *β* tubulin (green), actin (red), and nuclei (DAPI, blue). (c) Actin filament length quantification after 24, 48, 72, or 96 hours of exposure to 1 g (CTR) or s-microgravity (RPM) and (d) cell height measurements in the same experimental conditions. (e) Representative Western blots and corresponding densitometric analyses of *β* tubulin and *β* actin proteins in H9C2 cells cultured at 1 g (CTR) or s-microgravity (RPM). The densitometric analyses are plotted as the ratio between the optical density (OD) × mm^2^ of each band and OD × mm^2^ of the corresponding GAPDH band. Data in (c–e) are expressed as the mean ± SEM from three independent experiments. ^∗∗^*p* < 0.01*vs.* CTR; ^∗∗∗^*p* < 0.001*vs.* CTR.

**Figure 2:**
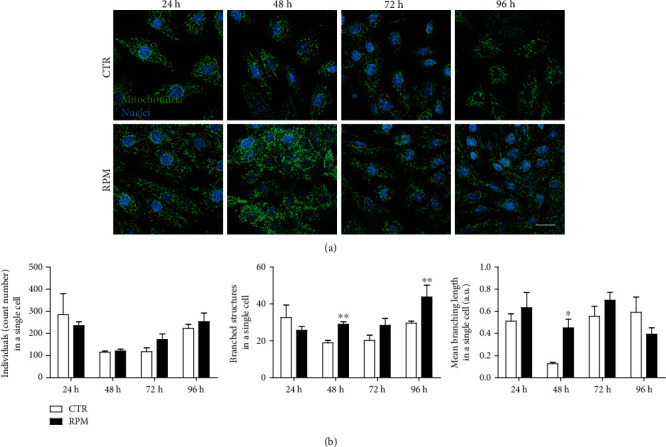
Mitochondria network. (a) Representative images of H9C2 cells at 1 g (CTR) or s-microgravity (RPM) stained with the translocase of the outer mitochondria membrane TOM20 (green), depicting the mitochondria morphology, and DAPI (blue), a specific dye for nuclei. Scale bar 20 *μ*m. (b) Quantification of mitochondria network morphology represented by these parameters: “individuals,” defined as the counts of the number of objects in the image that do not contain a junction pixel—it determines the number of punctuate or rod-shaped mitochondria, “branched structures in a single cell” outlined the number of objects in the image that contain at least 1 junction pixel and are thus comprised of more than one branch—it defines the number of mitochondria networks inside the cell, and “mean branching length in single cell,” the average length of all rods/branches per single cell. Data in (b) are expressed as the mean ± SEM from three independent experiments. ^∗^*p* < 0.05*vs.* CTR; ^∗∗^*p* < 0.01*vs.* CTR.

The H9C2 cells were grown in adhesion under standard (at 1 g, CTR) or s-microgravity (on RPM, RPM) conditions up to 96 hours to simulate a long-lasting exposure. At selected times (24, 48, 72, and 96 hours), cell morphological aspects were assayed. Figures 1 and 2 collect representative images and quantitative analyses from cell samples stained to depict cytoskeleton organization (actin and tubulin) and mitochondria networking, respectively. The quantitative analyses were performed on single optical section images (five images acquired from each sample from three independent experiments) acquired by a confocal microscope and analyzed using ImageJ software applications to quantify the mean length of actin or tubulin filaments present in single cells, in samples stained with Phalloidin and anti-*β* tubulin (Figure 1(a)), and to assess mitochondrial networking in single cells, in samples stained with TOM20 (Figure 2(a)) [27]. Observing the image collections of single optical sections, no striking difference came out between exposed and nonexposed cells (Figures 1(a) and 2(a)), excepting a slight change in the cytoskeleton structure (Figure 1(a)). Quantitative analyses showed that s-microgravity modulated the mean length of actin filaments and its effect became statistically significant at 96 h exposure time, while tubulin showed no significant variation in any tested condition (data not shown) (Figure 1(c)). In addition, Z-stack images were acquired and orthogonal (XZ) reconstruction was performed to allow the quantification of cell height (Figures 1(b) and 1(d)). The data shown in Figure 1(d) revealed that the s-microgravity deeply affected cell height at 24 and 48 hours of exposure (Figure 1(d)). The expression levels of the main components of the cytoskeleton (*β* tubulin and *β* actin) were not significantly modified in any tested condition (Figure 1(e)).

Concerning the quantification of mitochondrial networking, the following parameters were considered: individuals, branched structures, and branching length (Figure 2(b)) [27]. Data suggested that s-microgravity exposure affected mitochondrial branching complexity at 48 h and 96 h (Figure 2(b)).

### 3.2. Cell Proliferation and Cell Cycle

**Figure 3:**
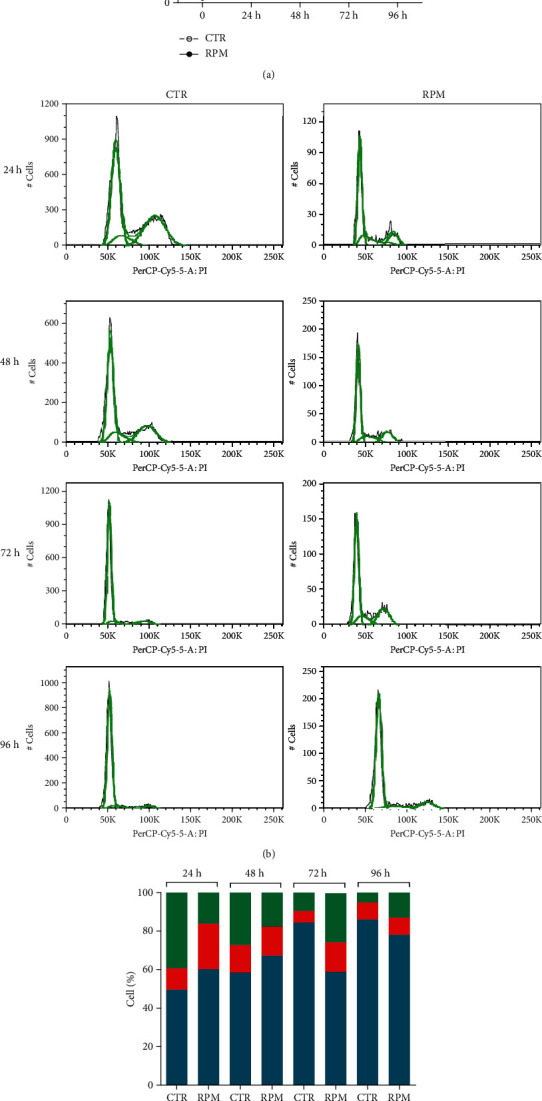
Cell proliferation and cell cycle. (a) Cell proliferation assay performed by live cell counting using trypan blue dye solution. Data are expressed as the mean ± SEM from three independent experiments. ^∗^*p* < 0.05*vs.* CTR; ^∗∗^*p* < 0.01*vs.* CTR. (b) Representative traces of flow cytometry analysis and (c) percentage of cell cycle phase distribution in s-microgravity (RPM) or 1 g (CTR) exposed cells at selected times (24, 48, 72, or 96 hours). The graph in (c) is a representative of three independent experiments.

During the period of exposure to s-microgravity, the H9C2 cells showed a slower proliferation rate within 48 hours in comparison with the control cells and then regained the normal growth progression (Figure 3(a)). Accordingly, the cell population showed an increase of cell percentage in the s-phase of the cell cycle after 24 hours of exposure, restoring the normal distribution after 96 h of exposure in comparison to the control populations (Figures 3(b) and 3(c)). Trypan blue exclusion test and cytofluorimetric analyses also showed no significantly detectable necrotic or apoptotic cells in both control and exposed cells.

### 3.3. Cellular Metabolic Status

**Figure 4:**
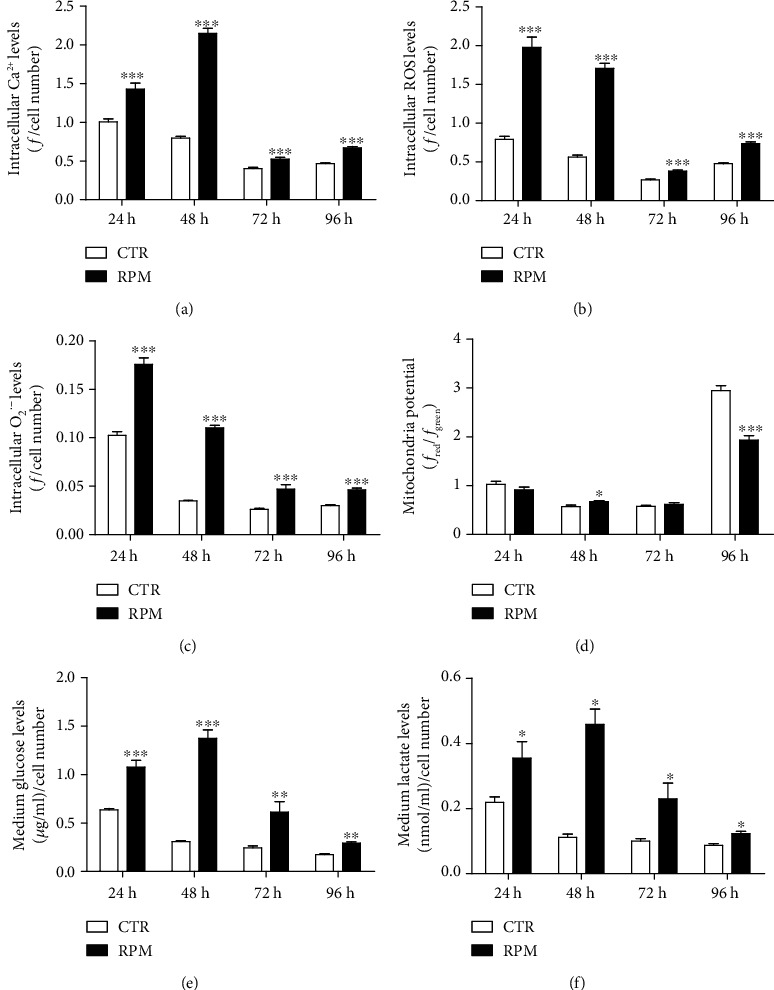
Cellular metabolic status. (a–c) Intracellular Ca^2+^, ROS, and O_2_^·−^ levels, respectively. Data are expressed as the mean ± SEM of *f*/cell number, where *f* is the fluorescence value acquired and normalized to the number of cells in the well. (d) Mitochondrial membrane potential expressed as the mean ± SEM of the red/green JC-1 fluorescence ratio. The results in (a, d) came from three independent experiments. (e) Glucose concentration (*μ*g/mL) and (d) lactate concentration (nmol/mL) in culture medium, respectively. Data are normalized to the number of cells and expressed as the mean ± SEM from three independent experiments. ^∗^*p* < 0.05*vs.* CTR, ^∗∗^*p* < 0.01*vs.* CTR, and ^∗∗∗^*p* < 0.001*vs.* CTR.

Some hallmarks of cell activity were measured to draw a picture of the metabolic status of the H9C2 cells. After exposure to s-microgravity, the cells showed significantly increased levels of intracellular Ca^2+^ (Figure 4(a)) and concomitant increases of intracellular ROS (Figure 4(b)) and mitochondrial O_2_^·−^ (Figure 4(c)); probably these increases contributed to the changes of mitochondrial membrane potential observed at different extent during the exposure (Figure 4(d)) in comparison to control cells. The change in metabolic activity of exposed H9C2 cells was also indirectly confirmed by measuring glucose and lactate levels in the cell medium. Both molecules resulted increase in the medium of s-microgravity-exposed cells at all times tested (Figures 4(e) and 4(f)). These results gave evidence for a decreased consumption of glucose and an increased production of lactate in exposed cells in comparison to control ones.

### 3.4. Early Times of Exposure to s-Microgravity

**Figure 5:**
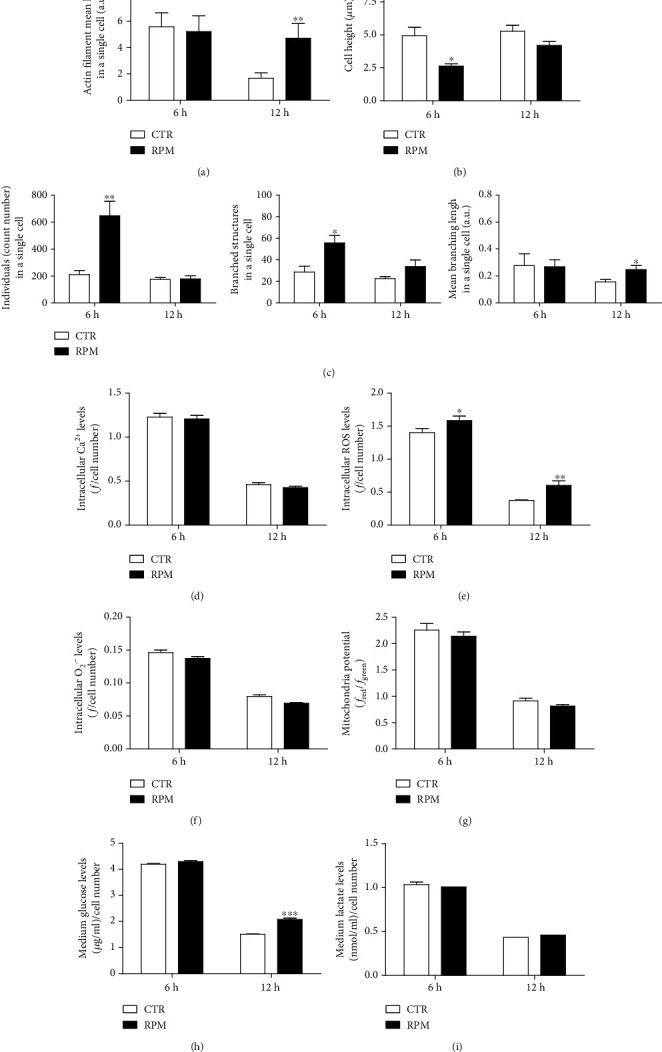
Effects of 6 h or 12 h exposure to s-microgravity. (a) Actin filament length evaluation in cells at s-microgravity (RPM) or at 1 g (CTR). (b) Cellular height measurements. (c) Mitochondria morphology measurements. (d–f) Intracellular Ca^2+^, ROS, and O_2_^·−^ levels, respectively. (g) Mitochondria membrane potential measurements. (h, i) Glucose and lactate concentrations in culture medium, respectively. Data are expressed as in Figures 1, 2, and 4 and are the mean ± SEM from three independent experiments. ^∗^*p* < 0.05*vs.* CTR, ^∗∗^*p* < 0.01*vs.* CTR, and ^∗∗∗^*p* < 0.001*vs.* CTR.

The most significant results showed that the s-microgravity-induced effects started from 24 h exposure time, so further earlier times were tested. The main morphological and metabolic features were assayed after 6 h and 12 h exposure times. The actin filament length resulted greater in 12 h exposed cells, the mean height lower at 6 h exposed cells, and mitochondrial networking was affected in 6 h exposed cells in comparison to each corresponding control (Figures 5(a)–5(c)). The intracellular Ca^2+^ levels did not appear modified in 6 h and 12 h exposed cells, while intracellular ROS levels resulted higher starting from the 6 h exposure time (Figures 5(d) and 5(e)). The O_2_^·−^ levels and mitochondrial membrane potential were not significantly different between 6 h and 12 h exposed cells and the corresponding controls (Figures 5(f) and 5(g)). Regarding glucose and lactate contents in the medium, the former resulted higher after 12 h exposure to s-microgravity; the latter did not change in exposed cells in comparison to controls (Figures 5(h) and 5(i)).

### 3.5. Strategies to Counteract the s-Microgravity-Induced Effects

**Figure 6:**
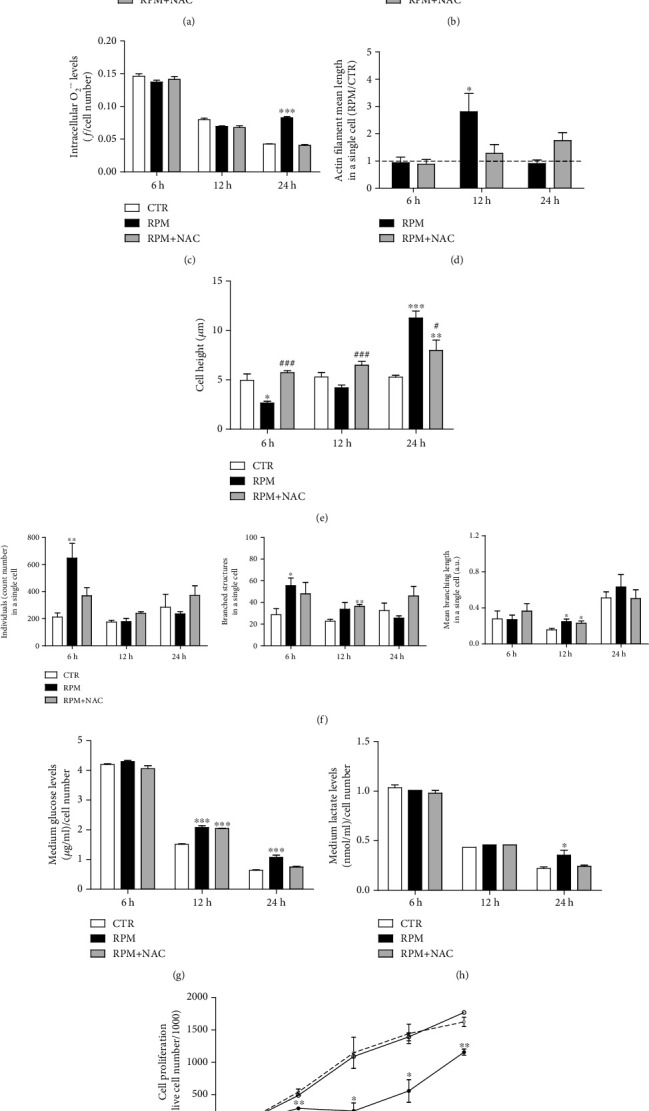
N-Acetyl-cysteine counteracts s-microgravity alterations in H9C2 cells. (a–c) Intracellular ROS, Ca^2+^, and O_2_^·−^ levels, respectively, in cells at s-microgravity (RPM) or at 1 g (CTR) in the absence or presence of NAC (+NAC). (d) Actin filament length evaluation. (e) Cellular height measurements. (f) Mitochondria morphology measurements. (g, h) Glucose and lactate concentrations in culture medium, respectively. (i) Cell proliferation. Data are expressed as in Figures 1–4 and are the mean ± SEM from three independent experiments. ^∗^*p* < 0.05*vs.* CTR, ^∗∗^*p* < 0.01*vs.* CTR, ^∗∗∗^*p* < 0.001*vs.* CTR, and ^###^*p* < 0.001*vs.* RPM.

The results described above suggested that after early 6 hours of exposure, the first metabolic sign affected by s-microgravity was represented by the increase of intracellular ROS levels. This suggests the presence of an unbalance of the oxidative metabolism. To counteract this effect and to verify if this is the *primum movens* of the effects observed at longer times of exposure, H9C2 were exposed to s-microgravity on RPM in the presence of 1 mM N-acetyl-cysteine (NAC), an antioxidant agent. The presence of NAC reestablished the ROS levels starting from the 6 h exposure time, as expected, and the intracellular Ca^2+^ and O_2_^·−^ levels at 24 h exposure time (Figures 6(a)–6(c)).

The antioxidant completely or at least partially prevented the effects induced by s-microgravity on cell shape and mitochondria networking (Figures 6(d), 6(e), and 6(f)) as well as on glucose and lactate contents in the medium (Figures 6(g) and 6(h)). Interestingly, the presence of NAC succeeded in restoring cell proliferation rate in exposed H9C2 cells (Figure 6(i)).

**Figure 7:**
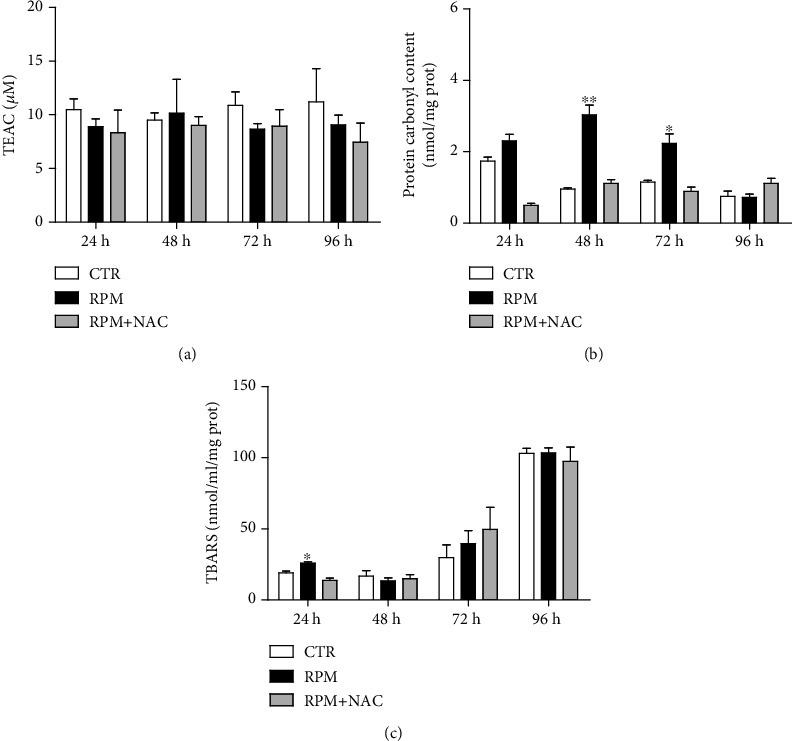
Oxidative damage markers. (a) TEAC (Trolox equivalent antioxidant capacity) value, expressed as *μ*M Trolox present in the sample mixture (see Materials and Methods). (b) Protein carbonyl content, expressed as nmol/mg proteins. (c) Thiobarbituric acid reactive substances (TBARS) expressed as nmol/mL/mg proteins. CTR: H9C2 cells were exposed to 1 g; RPM: H9C2 cells were exposed to s-microgravity; RPM+NAC: H9C2 cells were exposed to s-microgravity in the presence of 1 mM N-acetylcysteine. Data are expressed as the mean ± SEM from three independent experiments. ^∗^*p* < 0.05 vs. CTR; ^∗∗^*p* < 0.01 vs. CTR.

To evaluate the overall impact of s-microgravity at long exposure times on the cellular oxidative balance and the presence of oxidative damage markers, the total antioxidant status (tested by the Trolox equivalent antioxidant capacity (TEAC)), protein carbonyl content, and thiobarbituric acid reactive substances (TBARS) were assayed. The TEAC was not altered in any of the tested samples (Figure 7(a)). The increased ROS induced by s-microgravity exposure triggered a significant increase of protein carbonyl content and TBARS, markers of protein oxidation and lipid peroxidation, respectively (Figures 7(b) and 7(c)). In particular, the increase of protein carbonyl content in exposed cells became statistically significant after 48 and 72 hours, whereas after 96 h there was not any difference between exposed and control cells (Figure 7(b)). The lipid peroxidation resulted significantly increased in 24 h exposed cells in comparison to control ones (Figure 7(c)). The presence of NAC prevented also these effects (Figures 7(b) and 7(c)).

## 4. Discussion

A large number of experimental evidences pointed out that single cells react to changes in gravity and that this reaction might play a crucial role for physiological modifications at the organ level during space flight. The microgravity environment during space flights or exposure to weightlessness as during staying on board the ISS is a potential source of cardiovascular changes. Even if numerous evidences regarding cardiovascular alterations, such as an increase in the risk of ventricular arrhythmias and the reduction in cardiac muscle mass and function, are reported, less is known about the alteration at the cellular level [29–31].

In this study, we investigated the effect of s-microgravity at the cellular level in cardiomyocyte-like cells, looking for potential changes on their morphology, cytoskeleton, and metabolism.

To this purpose, we used the RPM, a bench top microgravity simulator, also previously used to expose other cell phenotypes [22, 23, 32], and widely accepted as a valid tool for on-ground studies concerning cell exposure to a weightless environment [33, 34]. Usually, due to its principles of function mode, the RPM is used to investigate the effect of s-microgravity on slow processes (on the timescale of hours) and to assay possible detrimental effects or adaptation processes [34]. Thus, using this microgravity simulator, it is not possible to reveal early alterations on the order of seconds or minutes induced by the s-microgravity.

In H9C2 cardiomyocytes, we did not observe striking qualitative differences in microtubules or actin organization after s-microgravity exposure. Quantitative analyses revealed a significantly increased mean actin filament length at early 12 h and late 96 h exposure times. However, if one considers also that the expression levels of cytoskeleton proteins are not modified after exposure, this suggested that the overall microtubules and microfilaments appeared to retain their overall organization.

Many and apparently conflicting results were published concerning the microgravity-induced effects on the cell cytoskeleton, and these can be related to cell phenotype, exposure time, and real or simulated microgravity. Vorselen and colleagues reviewed the results obtained from studies concerning the effects of changes in gravity force on the cell cytoskeleton [35]. They concluded that, even if at a different extent and with different features in mammalian cells, the cytoskeleton could be considered the initial gravity sensor that in turn triggers intracellular responses [35].

Indeed, in primary cardiomyocytes exposed to real microgravity (Shenzhou-6 mission), alterations of microtubule assembling were reported just after 4 h after launching, while microfilaments (actin) did not show significant changes in distribution and organization [36]. In the same cardiomyocyte phenotype exposed to on-ground clinorotation-simulated microgravity, the microfilaments and microtubules were found disorganized in a distribution with no significant depolymerisation [37]. The different peculiar behaviour of the cell cytoskeleton could be due to different conditions between real and simulated microgravity and to different time exposure, as also proposed by other authors [36, 38]. In our model, the increased mean actin filament length observed at early 12 h and late 96 h exposure times suggested an s-microgravity sensitive actin network that could indicate a dynamic response of actin microfilaments that could cause transient changes in cell height at early (decreased height after 6 h exposure) and later (increased height after 24 and 48 exposures) times. A similar dynamic behaviour was observed after exposure to RPM-generated s-microgravity also in other cell phenotypes such as TCam-2 seminoma cells and MC3T3-E1 osteoblast-like cells [22, 32]. Once more, ours and other published results on structural modifications on cytoskeletal components in response to the microgravity environment suggest a cell type-specific response, which could be explained, at least in part, by the different cytoskeletal dynamics and regulation of actin architecture and microtubule organization [39–42]. It is well known that the influence of the external environment on the cytoskeleton structure can be of great importance in affecting cellular functionality [43]. The cytoskeleton consists of different components: microtubules built by tubulin, microfilaments of actin, and intermediate filaments. Many authors proposed a functional role for the cytoskeleton network that is recognized such as a modulator of cell biological features as morphology, motility, mitosis, intracellular organelle compartmentalization, and thus metabolism [44–46]. The resulting extracellular forces on the cytoskeleton scaffold have a pivotal role in influencing the proliferation and the cell cycle [47]. In our experimental conditions, starting after the first 24 h of cell exposure, we observed a reduction in cell proliferation rate and cell accumulation in S-phase of the cell cycle; even if at longer exposure times, the cells appeared to rescue the normal replication rate, indicating that in H9C2 cells, the s-microgravity-induced effect is transient. The microgravity-induced decrease in cell proliferation was also seen in other cell phenotypes [22, 32, 48, 49]. However, there are controversial evidences about the effects of microgravity on cell growth in stem cells and cardiomyocytes differentiating from pluripotent stem cells [50–54]. These introduce the hypothesis that the effects induced by microgravity are not only phenotype-dependent but also dependent on the functional and metabolic status of the cells tested.

In our H9C2 cardiomyocytes, s-microgravity induced also metabolic changes. Indeed, s-microgravity exposure affected the mitochondria arrangement, intracellular Ca^2+^ levels, and cell oxidative status. In particular, the s-microgravity-induced increases of Ca^2+^and ROS (including the species produced by mitochondria) levels were complemented by a possible increased anaerobic activity (lower glucose uptake and higher lactate production) and lasted up to 96 h long-lasting exposure. It was reported that in cardiomyocytes mitochondrial dysfunction and ROS production ue to microgravity exposure, if not properly prevented, could have a pivotal role in guiding the modifications that lead to cardiac dysrhythmias, as observed in some astronauts [55].

In our study, at early exposure times (6-12 h), the features affected by s-microgravity were ROS production, actin and mitochondria network arrangement, and glucose uptake.

Indeed, one of the first alterations due to the exposure to s-microgravity (just after 6 hours) is linked to ROS imbalance, while intracellular Ca^2+^appeared to be modified later (starting after 24 h of exposure). The timing of the effects induced by s-microgravity suggested that the early ROS increase and cytoskeletal alterations play as main actors of the “mechano-chemo-transduction.” In addition, the increased ROS affected the proteins of the L-type calcium channels and ryanodine receptors [56]; thus, we can hypothesize that this could be the event triggering the intracellular Ca^2+^ rise.

Interestingly, the cell height and actin filament length were promptly modified at the early time of exposure to s-microgravity. There is a large body of evidence reporting that mechanical loading plays a critical role in the rearrangements of the cytoskeleton thus influencing in turn also cell growth, metabolism, and differentiation [57–59]. These evidences support the hypothesis that in H9C2 cardiomyocytes the s-microgravity, by altering external force distribution, triggers cell responses through a cytoskeleton mechanotransduction mechanism and ROS production. Interestingly, the use of the antioxidant NAC prevented almost completely the effects induced by s-microgravity exposure at early and late times. Similar results were observed also in other cell models [22, 32, 60].

It is evident from our results that the presence in the culture medium of the antioxidant NAC during exposure to s-microgravity abolished its effects on cell cycle progression, thus linking these alterations to the oxidant production. Even if the generation of ROS in cellular physiology is a normal part of metabolic homeostasis, oxidative stress and damage can be the result of cellular dysregulation of the physiological balance between free radicals' production and scavenging. Space travels and habitation in reduced gravity require the implementation of countermeasures able to prevent these alterations. In this context, some antioxidant strategies appear to be efficacious. In an experiment flown to the International Space Station in 2017, nanoceria was used to counteract oxidative stress in muscle cells [61].

In this study, we observed that s-microgravity deeply affected the metabolic status of H9C2 cells, increasing the lactate levels in the extracellular media. This could be due to a direct alteration of mitochondria activity by ROS unbalance. Indeed, treatment with the antioxidant NAC, acting as ROS scavenger, reverted this effect, avoiding ROS-induced damage in H9C2 cells. These results are in accordance with the reduction in aerobic capacity observed in astronauts [62] that could be due to the lack of control on ROS overproduction, although the molecular mechanisms are still not fully understood.

We observed not only increased ROS production but also extracellular secretion of lactate and a lower use of glucose that, in turn, are indicative of a change in the metabolic status of the cells. It is of note that also in our cardiac-like cells the exposure to s-microgravity significantly influenced the cell energy capacity and the capability of coupling oxygen consumption with oxidative phosphorylation. This is in line with other results obtained in another muscular model like C2C12 cells [63] and in a bone model in which metabolomics and proteomic analyses revealed increased glycolysis, while the Krebs cycle was blocked at succinate-fumarate transformation after s-microgravity exposure [64]. Although it is still necessary to find all pieces of the puzzle that draws the correlation between simulated microgravity and metabolic effects, certainly the excess of ROS and mitochondrial functionality appear as promising key candidates. These results, added to data obtained also in other models, improve the knowledge regarding the effects induced by microgravity on the oxidative status of the cells and suggest a possible antioxidant protective strategy.

Although further experiments and trials are needed, the NAC detoxifying effect on early ROS rise observed in this study suggests a possible countermeasure to be adopted in the planning of the diet for astronauts even before the space missions.

## 5. Conclusions

**Figure 8:**
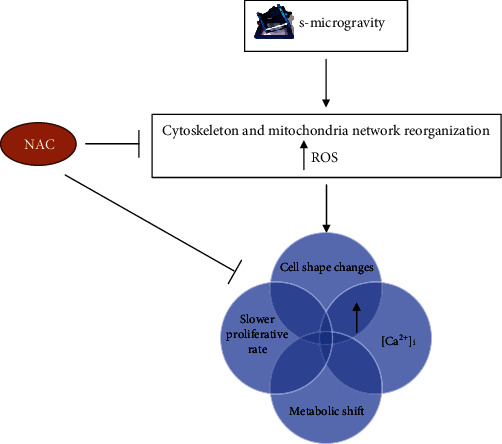
Scheme of the essential effects induced by s-microgravity exposure on H9C2 cardiomyocytes.

The use of RPM and H9C2 cardiomyocytes made it possible to delineate the effects triggered by the exposure to s-microgravity and hypothesize a mechanism of action. Compared to cell grown at earth gravity, we found a slower cell proliferation rate and lower metabolic activity in s-microgravity exposed H9C2 cells. Indeed, after RPM exposure, H9C2 cells showed cytoskeletal and mitochondria network rearrangements, increased levels of intracellular ROS, [Ca^2+^]_i_ and mitochondrial superoxide anion. The increase of oxidant species was followed by protein carbonyl formation. In an overview (Figure 8), our data suggest that, in a cardiomyocyte-like model, s-microgravity early affects the cell cytoskeleton that acts as a mechanosensor and a concomitant increase of intracellular ROS levels. The alterations in oxidative balance could, in turn, drive all other observed effects resulting in an alteration of cell metabolism and thus a slower proliferative rate and protein carbonyl formation, implying a possible alteration in the functional efficiency of proteins as those involved in the excitation-contraction mechanism. Interestingly, the presence of NAC and its antioxidant capacity reverted the effects induced by s-microgravity.

These data suggest that an appropriate antioxidant supplementation could prevent also cardiac dysfunction induced by human permanence in microgravity conditions.

## Figures and Tables

**Table 1:** Fluorescent probes, used for intracellular analyses.

Probe	Excitation (nm)	Emission (nm)	Analyses
Fluo4-AM5 *μ*M	488	520	Intracellular Ca^2+^ levels
H_2_-DCFDA10 *μ*M	488	520	Intracellular ROS levels
MitoSox RED 5 *μ*M	510	580	Mitochondrial O_2_^−^ levels
JC1 5 *μ*g/mL	488	520/590	Mitochondrial membrane potential

## Data Availability

All data are available on demand.
